# The association of enzymatic and non-enzymatic antioxidant defense parameters with inflammatory markers in patients with exudative form of age-related macular degeneration

**DOI:** 10.3164/jcbn.16-30

**Published:** 2017-02-08

**Authors:** Emina Čolak, Svetlana Ignjatović, Aleksandra Radosavljević, Lepša Žorić

**Affiliations:** 1 Institute of Medical Biochemistry, Clinical Center of Serbia, School of Pharmacy, University of Belgrade, Belgrade 11000, Serbia; 2 Institute of Ophthalmology, Medical Retina Department, Clinical Center of Serbia, School of Medicine, University of Belgrade, Belgrade 11000, Serbia; 3 Clinic for Eye Diseases, Clinical Center, Faculty of Medicine, Settlement Kosovska Mitrovica, Kosovska Mitrovica 38200, Serbia

**Keywords:** age-related macular degeneration, oxidative stress, antioxidants, inflammation

## Abstract

There are evidence that oxidative stress and inflammation are involved in the pathogenesis of the age-related macular degeneration (AMD). The aim of this study was to analyze the antioxidant defense parameters and inflammatory markers in patients with exudative form of AMD (eAMD), their mutual correlations and association with the specific forms of AMD. The cross-sectional study, included 75 patients with the eAMD, 31 patients with the early form, and 87 aged-matched control subjects. Significantly lower SOD, TAS and albumin values and higher GR, CRP and IL-6 were found in the eAMD compared to the early form (*p*<0.05). Significant negative correlations were found between GPx and fibrinogen (*r* = –0.254), TAS and IL-6 (*r* = –0.999) and positive correlations between uric acid and CRP (*r* = 0.292), IL-6 and uric acid (*r* = 0.398) in the eAMD. A significant association of CRP (OR: 1.16, 95% CI: 1.03–1.32, *p* = 0.018), fibrinogen (OR: 2.21, 95% CI: 1.14–4.85, *p* = 0.021), TAS (OR: 7.45, 95% CI: 3.97–14.35, *p* = 0.0001), albumin (OR: 1.25, 95% CI: 1.11–1.41, *p* = 0.0001) and uric acid (OR: 1.006, 95% CI: 1.00–1.02, *p* = 0.003) was found with the eAMD. In conclusion it may be suggested, there is a significant impairment of antioxidant and inflammatory parameter levels in eAMD patients. In addition, significant association exists between the tested inflammatory markers and antioxidant parameters with late-eAMD.

## Introduction

Age-related macular degeneration (AMD) is the leading cause of irreversible central visual loss among the elderly in the developed countries. The prevalence of early form of AMD is 18% in the population of 65 to 74 years of age, rising to 30% after 74 years of age.^(1)^ Aging is associated with biological changes in the eye, including cumulative oxidative injury. Free radicals are constantly synthesized and involved in a series of toxic effects such as lipid peroxidation, protein and DNA oxidative modifications.^(1)^ Vision loss in AMD occurs through photoreceptor damage in the macula, with abnormalities in the retinal pigment epithelium (RPE) and Bruch’s membrane.^(2)^ Excessive exposure to light is associated with age-related macular degeneration.^(3)^ *In vivo*, lipofuscin granules in the RPE are continually exposed to visible light (400–700 nm) and high oxygen tensions (70 mmHg), ideal conditions for the formation of the reactive oxygen species, with the potential to damage cellular proteins and lipid membranes. It has been hypothesized that the photosensitization reactions may be involved in the development of AMD, via synthesis of the reactive oxygen species such as: superoxide, hydrogen peroxide, and singlet oxygen, which may damage the RPE and Bruch’s membrane.^(4)^

Different types of the oxidants produced in cells require that cells have different types of the antioxidant defense parameters.^(5,6)^ The major cellular antioxidants are enzymatic substances such as superoxide dismutase (SOD), Se-dependent glutathione peroxidase (GPx), catalase (CAT), glutathione reductase (GR) and various non-enzymatic low molecular substances such as: glutathione, retinoids, carotenoids, ascorbic acid, vitamin E, albumin, uric acid, bilirubin, transferrin, ceruloplasmin, which act as various scavengers for different types of reactive oxygen species.

Aging is associated with higher frequency of several disorders including the atherosclerosis, peripheral vascular disease, coronary artery disease, type 2 diabetes mellitus, dementia, Alzheimer’s disease, etc.^(7,8)^ Aging is also characterized by proinflammatory state that contributes to the onset of disability in age-related diseases. Over the last two decades, a prominent role of inflammation in the pathogenesis of AMD has been established. Lipid accumulation in RPE cells combined with oxidative stress over time results in the formation of lipid peroxidation products such as 4-hydroxynonenal (4-HNE), malondialdehyde (MDA), isoprostanes (F_2_-IsoPs), acrolein, hexanoyl-lysine, and oxidatively modified low-density lipoprotein (ox-LDL) which have cytotoxic and pro-inflammatory properties on RPE cells.^(9)^

The aim of this study was to analyze the activities of antioxidant enzymes: SOD, GR, and GPx, along with the non-enzymatic low molecular substances (albumin, uric acid and bilirubin) and the Total Antioxidant Status (TAS) of serum, as well as the acute inflammatory markers (CRP, IL-6 and fibrinogen) in patients with AMD. In addition, the aim was to correlate these parameters in relation to the different stages of AMD defined as the early and exudative-advanced form of AMD, in order to find the possible impact of tested parameters to the development of specific forms of the disease.

## Materials and Methods

### Patients

In the cross-sectional study, conducted at the Clinic of Ophthalmology, University of Belgrade, out of 106 patients with the age-related macular degeneration with mean age of 71.3 ± 7.04 years, and 87 age matched control subjects comprising the control group (CG), were included in the study The patients underwent complete ophthalmological examination including the visual acuity assessment, color fundus photography and fluorescein angiography. They were thoroughly clinically examined and fulfilled questionnaire about their habits including the BMI, physical activity, smoking, etc. One of the pathological hallmarks of AMD is the focal deposition of the extracellular material between the retinal pigment epithelium (RPE) and Bruch’s membrane called drusen, visualized as yellow deposits under the retinal pigmented epithelium. The Age-Related Eye Disease Study (AREDS) defined categories based on the exam findings of drusen, atrophy, and neovascularization, these categories are defined as: a) no AMD (fewer than 5 small drusen, <63 µm), b) mild AMD (multiple small drusen or some intermediate sized drusen, 63–124 µm), c) intermediate AMD (extensive intermediate sized drusen, more than one large, >125 µm, or non-central geographic atrophy), and d) advanced AMD, with two subcategories: central geographic atrophy (also known as “advanced-dryˮ AMD) and choroidal neovascularization (the creation of a new blood vessels in the choroid layer, causing vision loss in one eye) also known as “wet” or advanced-exudative form of AMD.^(10)^ This classification was based on most severely affected eye. Our interest categories were those with advanced-exudative AMD, and “early” form of AMD (mild and intermediate).

The AMD patients were not receiving any anti-VEGF therapy. The exclusion criteria for patients and control subjects were: the presence of any other ocular neovascular disease such as glaucoma, cataracta, chronic uveitis, intra or extra ocular tumors, or the presence of some systemic disease like rheumatoid arthritis, cardiovascular disease, Thyroid disease inflammatory bowel disease, spondyloarthropathy synovitis, tuberculosis and malignant tumors.

The subjects in the control group were recruited from the employees of the Institute of Ophthalmology, CCS, Belgrade, and their relatives, who were without any signs of the acute conditions or maculopathy at the time of the study.

All subjects gave their informed consent on participation in the study, and the local Ethics Committee approved this study. This study was performed according to the Declaration of Helsinki.

### Methods

The blood samples for analysis were taken after 12–14 h of the overnight fast. All laboratory tests were done immediately. Antioxidant parameters SOD, GPx, GR and TAS were determined by commercial tests Randox Ltd. UK, based on spectrophotometer methods, according to Goldstein for SOD, Paglia for GPx, Müler for TAS, and Goldberg for GR.^(11–14)^

SOD was determined in blood hemolysate, which was obtained by washing of the erythrocytes 4 times with 3 ml of 154 mmol/L NaCl and finally by lysing of the washed erythrocytes with cold deionized water and leaving it in place 15 min at +4°C to complete the hemolysis.

GPx was determined in the whole blood sample, which was, just before determination, diluted 41 times by gradual addition of diluent (supplied in the test kit) and double-concentrated Drabkin’s reagent.

TAS and GR were determined in plasma that was obtained by centrifugation of the Li-heparinized blood 10 min/3,000 rpm.

The analytical accuracy and precision were tested according to the manufacturer’s protocol, using the control materials provided by the manufacturer. The within run inprecision (CV%) for SOD was 4.7%, for GPx was 4.5%, for GR 3.2% and for TAS was 2.3%, and the between run inprecision for SOD was 5.9%, for GPx 7.3%, for GR 5.2% and for TAS was 4%.

CRP was determined by an immunochemical high sensitive (hsCRP) method using the Olympus AU 400 analyzer while fibrinogen was determined by Clauss method on Behring Coagulation System XP analyzer. IL-6 was determined by means of chemiluminescent method on Access (Beckman-Coulter) immunochemical analyzer. CRP and IL-6 were measured in serum, while fibrinogen was determined in citrated plasma.

Albumin, uric acid and bilirubin concentration were determined in serum, using standard laboratory methods, on Olympus AU 400 biochemical analyzer.

### Statistical analysis

Statistical analysis was performed by MedCalc ver. 9.4.2.0 statistical package using the Student’s *t* test, Mann-Whitney *U* test, Chi-Square, ANOVA and Kruskal-Wallis test. Results were presented as mean ± SD for continuous normally distributed variables, and as median and interquartile range for non-normally distributed data. Spearman’s rank and Pearson’s correlation test was used to define correlations of the individual parameters between and within tested groups. All statistical tests were two-tailed. *P* values ≤0.05 were considered statistically significant. Linear regression and logistic regression analysis were used to model the association of the antioxidant and inflammatory markers to the advanced and early form of AMD.

## Results

The values of tested parameters and general information about the subjects were presented in Table 1.

According to AREDS classification, out of a total number of AMD patients, 75 had advanced exudative form of the disease-choroidal neovascularization (late AMD), out of whom 53 patients had only one eye and 22 patients had both their eyes affected. The rest of 31 patients had the early form of AMD. Out of a total number of studied AMD patients, 73.4% were females and 26.6% were males.

Lower SOD activity (*p* = 0.043) and TAS concentration (*p* = 0.0004), and higher GR activity (*p* = 0.04) were found in the AMD patients with the exudative form of disease compared to the early form of AMD (Table 1). Significantly higher CRP and IL-6 values were found in the same group of patients (*p*<0.05) compared to the early form. The fibrinogen values were elevated in both tested AMD groups compared to the controls (*p* = 0.007), but the average values were very similar in both, early and advanced AMD group (*p*>0.05).

The values of non-enzymatic antioxidant parameters differ significantly between the tested groups. Significantly lower albumin values (*p*<0.001) were obtained in AMD patients compared to the controls, in both early (*p* = 0.004) and exudative form of AMD (*p* = 0.05). Lower albumin values were recorded in exudative AMD group compared to the early form (*p* = 0.04). The uric acid values were also significantly lower in both tested subgroups compared to the control group (*p* = 0.044 and *p* = 0.048 respectively). No significant difference of uric acid values was obtained between the two subgroups of AMD patients (*p*>0.05). No significant difference was obtained between the two tested subgroups regarding the bilirubin values, nor comparing to the control group.

**Fig. 1:**
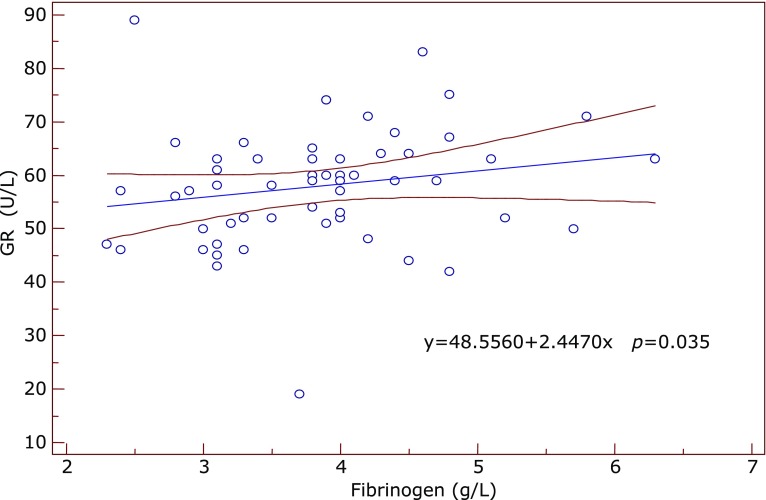
Positive correlation between GR (y axis) and fibrinogen values (x axis) in the group of early AMD patients. Regression line with 95% CI (confidence interval). Regression equation: y = 48.556 + 2.447x, *p* = 0.035.

**Fig. 2:**
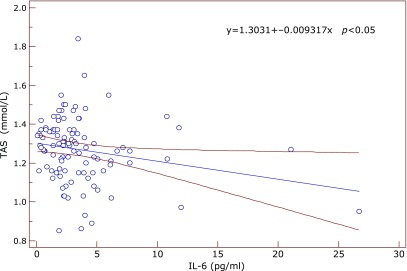
The negative correlation between TAS (y axis) and IL-6 (x axis) in the group of exudative AMD patients. Regression line with 95% CI (confidence interval). Regression equation: y = 1.3031 + –0.009317x, *p*<0.05.

**Fig. 3:**
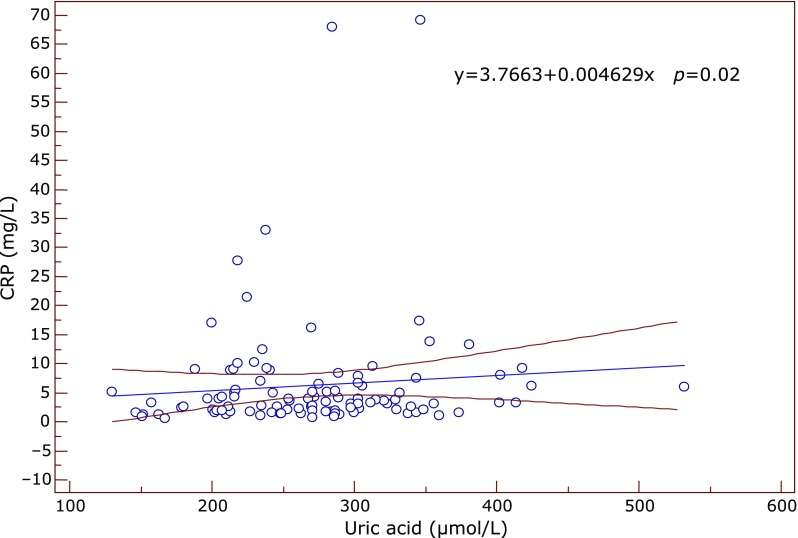
The positive correlation between uric acid (x axis) and CRP values (y axis) in the exudative AMD group. Regression line with 95% CI (confidence interval). Regression equation: y = 3.7663 + 0.004629x, *p* = 0.02.

**Fig. 4:**
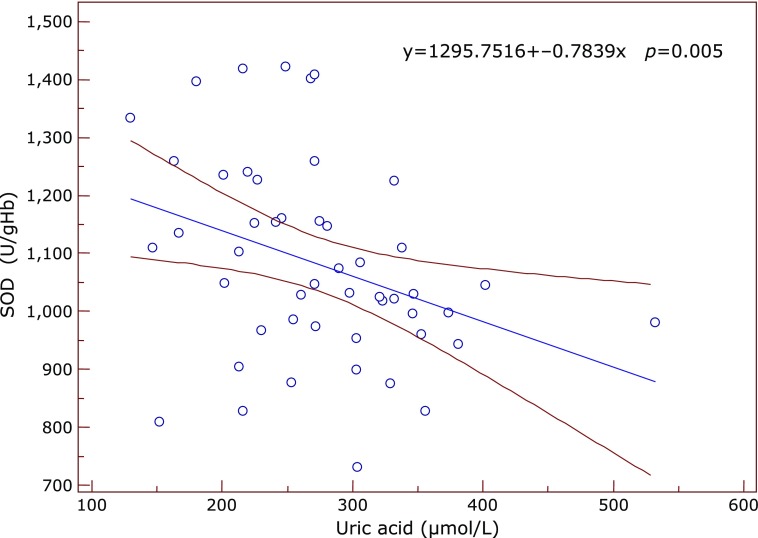
The inverse correlation between SOD (y axis) and uric acid (x axis) in the exudative AMD group of patients. Regression line with 95% CI (confidence interval). Regression equation: y = 1295.7516 + –0.7839x, *p* = 0.005.

Significant correlations between the inflammatory and antioxidant parameters were obtained by Spearman’s and Pearson’s correlation coefficient. Significant positive correlation between SOD and IL-6 was found in the subgroup of early AMD (ρ = 0.612, *p* = 0.05) at the borderline value of significance. In the same subgroup GR correlated positively with fibrinogen (ρ = 0.609, *p* = 0.035) (Fig. 1) while TAS correlated negatively with IL-6 (ρ = –0.633, *p* = 0.045) and with SOD (ρ = –0.568, *p* = 0.016). In the advanced-exudative AMD subgroup, GPx correlated negatively with fibrinogen (ρ = –0.254, *p* = 0.055), TAS with IL-6 (*r* = –0.999, *p* = 0.05) (Fig. 2) at the borderline value of significance, while uric acid correlated positively with CRP (*r* = 0.292, *p* = 0.02) (Fig. 3) and IL-6 (*r* = 0.398, *p* = 0.048). An inverse correlation was found between enzymatic and non-enzymatic antioxidants such as: SOD and uric acid (*r* = –0.401, *p* = 0.005) (Fig. 4), GPx and TAS (*r* = –0.346, *p* = 0.016) in the exudative AMD patients, and between SOD and TAS (*r* = –0.568, *p* = 0.016) in the early AMD subgroup.

A negative correlation was recorded between albumin and CRP (ρ = –0.339, *p* = 0.022) and between bilirubin and CRP at the borderline value of significance (ρ = –0.185, *p* = 0.05) in the whole group of AMD patients.

**Fig. 5:**
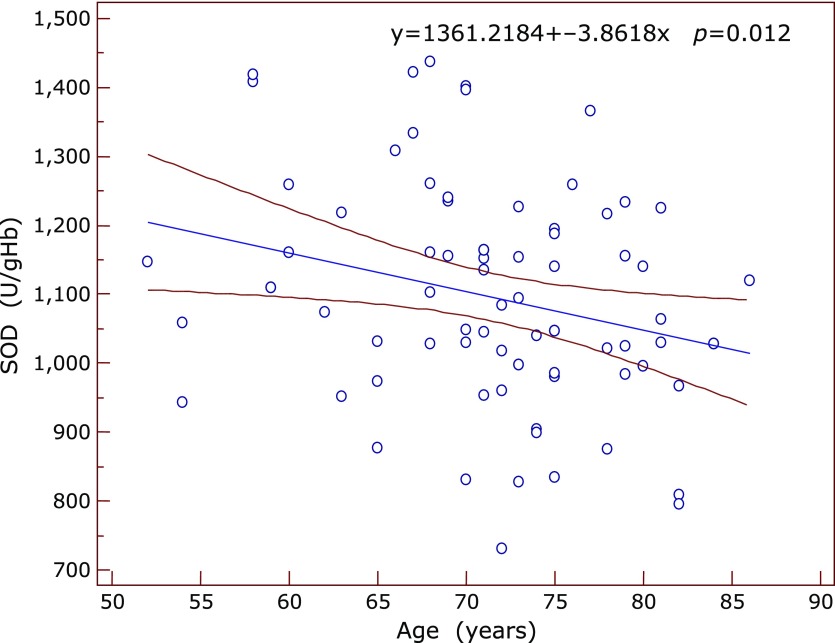
The negative correlation between SOD values (y axis) and patient’s age (x axis) in the whole AMD group. Regression line with 95% CI. Regression equation: y = 1361.2184 + –3.8618x, *p* = 0.012.

It is important to mention that a significant and weak negative correlation was obtained between SOD and the age of patients in the whole group of AMD (*r* = –0.285, *p* = 0.012) (Fig. 5), while a stronger correlation was found in the subgroup of exudative AMD (*r* = –0.405, *p* = 0.005). Very strong and negative correlation was recorded between the GPx and aging in the exudative form of AMD (*r* = –0.844, *p* = 0.017), while GR correlated positively with subject’s age in the same group of AMD patients (*r*=0.844, *p* = 0.039).

Using the logistic regression analysis, a significant association was obtained between occurence of advanced-exudative AMD and CRP values (OR:1.16, 95% CI: 1.03–1.32, *p* = 0.018), fibrinogen (OR: 1.77, 95% CI: 0.99–3.15, *p* = 0.05), TAS concentration (OR: 7.45, 95% CI: 3.97–14.35, *p*<0.001) albumin (OR: 1.25, 95% CI: 1.11–1.41, *p* = 0.0001) and uric acid (OR: 1.006, 95% CI: 1.00–1.02, *p* = 0.003) (χ^2^ = 27.3, *p* = 0.0003) (Table 2).

## Discussion

The obtained results support the hypothesis that AMD patients have a significant impairment of antioxidant defense system and inflammatory response in comparison to control group.

This study has documented that there is a significant difference in the values of antioxidant defense parameters between early and advanced-late AMD. Late form of AMD is associated with choroidal neovascularization, higher SOD, TAS, albumin and uric acid deficiency and higher degree of inflammation. The parameters of the systemic acute inflammation, CRP and IL-6, were higher in the late form of AMD in relation to these parameters in the early form of AMD.

A large number of correlation obtained between the studied parameters indicated a close relationship between the tested parameters, their mutual influence and connections with the pathogenic mechanisms that underlying this disease. There was a synergistic relation of the enzymatic antioxidants such as GPx and SOD in all tested subgroups and an inverse correlation between enzymatic and non-enzymatic antioxidants (GPx and TAS, SOD and TAS, SOD and uric acid) as well. The majority of examined antioxidants were negatively correlated with inflammatory markers (GPx and fibrinogen, TAS and IL-6, SOD and TAS, GPx and TAS, albumin and IL-6) indicated that the increase of inflammation was followed by a decreasing activity of antioxidant enzymes, except for GR and uric acid, whose concentration increased with increasing the degree of inflammation.

The increase of IL-6 value occurs with aging, which is documented in many studies (correlation found only in the control group) and is followed by lower activity of GPx.^(15,16)^ We also found a mutual positive correlation between the inflammatory markers in both groups (AMD and CG) (GR and fibrinogen) suggesting that these changes were associated with aging and not related to AMD. Increased inflammation and reduced antioxidant defense system is also a consequence of aging, but chronic inflammation, change of their usual relationship and appearance of some new correlations could be a consequence of disease (AMD).

Oxidative stress and oxidative damage play a significant role in several ocular diseases including the age-related macular degeneration, cataract, uveitis, corneal inflammation, keratitis, etc.^(16)^ The toxic effects of reactive oxygen species and other free radicals can be eliminated by specific antioxidant enzymes (SOD, GPx, CAT, etc.) which can help the cell to regain the pro-oxidant-antioxidant balance. More severe oxidative stress can cause cell death, apoptosis and necrosis. Behndig *et al.*^(16)^ reported lower activities of SOD isoenzymes in tears, cornea, sclera, aqueous humor, lens, with the highest activity in the retina. Imamura *et al.*^(17)^ reported that the lack of SOD_1_ (Cu,Zn-SOD) could accelerate age-related pathological changes in the human retina such as drusen, thickened Bruch’s membrane and retina neovascularization. The inflammatory reaction is an important source of the oxygen-free radicals. Large amounts of superoxide radicals are secreted by activated phagocytic leukocytes, and also formed as by-product during biosynthesis of leukotrienes and prostaglandins and formation of lipid peroxides.^(18)^ It has been documented that in the conditions of increased oxidative stress and generation of hydrogen peroxide, the SOD isoforms, EC-SOD and Cu,Zn-SOD, are susceptible to inactivation by blocking the enzyme’s active center.^(19)^ On the other hand, a specific aging-related reduction of Cu,Zn-SOD activity in human lens has been previously reported. Therefore, decreased activity of SOD could be a consequence of these activities and important marker of the advanced form of AMD. According to Indo’s “modified mitochondrial superoxide theory of oxidative stress”, the superoxide generated from mitochondrial plays an important role in oxidative stress related diseases and aging, and that mitochondrial MnSOD is essential antioxidant enzyme for maintenance of cellular resistance to oxidative stress.^(20)^

Using the logistic regression analysis, we showed that there was significant influence of some antioxidant markers (TAS, uric acid, albumin), and some inflammatory markers (CRP and fibrinogen) on the occurrence of advanced-exudative form of AMD.

Lower serum albumin concentration was documented by Virgolici *et al.*^(21)^ in patients with age-related cataract, indicated that, increased oxidative stress and decreased redox albumin capacity were closely associated with development of premature age-related cataract. Venza *et al.*^(22)^ analyzed the impact of antioxidant enzymes and products of oxidative modification to macromolecules on development of age-related macular degeneration in 308 patients. They concluded that ageing was closely associated with oxidative stress and antioxidant status of tested patients. An inverse relationship of tested oxidant and antioxidant parameters was recorded, and a positive association between the determined antioxidant enzymes as well.

Regarding the uric acid, previous epidemiological studies have shown that there was a positive correlation between serum concentrations of this parameter and the development of various diseases such as essential hypertension, metabolic syndrome, diabetes mellitus, etc., but so far, there were no data on how and whether aging influenced the serum uric acid levels.Test results of Horwath-Winter and associates showed that subjects with senile cataract had significantly lower values of this parameter in the aqueous humor and tears than in the serum,^(23)^ and that these values were significantly reduced in the course of the disease.In contrast to our results, Subramani *et al.*^(24)^ reported higher serum uric acid levels in AMD patients with the neovascular form of AMD (exudative AMD) compared to control group, and in relation to geographic atrophy, although in the overall group of patients with AMD, a mean value of uric acid did not differ significantly from the average values in the control group.

It is very important to mentioned the obtained negative correlation of patients age with the antioxidant enzymes (SOD and GPx) and positive correlation between GR and ageing, which is logical, given that SOD and GPx activities tended to decrease, while GR activity tended to rise in AMD patients, especially in the exudative form of disease.

Suzuki *et al.*^(18)^ found within macular photoreceptors and the RPE of normal eyes, increasing quantities of oxidized phospholipids with advanced age. Progressive accumulation of the undigested lipid peroxidation products will stress RPE, which can ultimately induce apoptosis, a well established process in aging and AMD.^(25)^ Oxidatively modified substances can stimulate the expression of gp130, the signal transducing chain of IL-6 receptor family and the secretion of IL-6.^(26)^ IL-6 induces proliferation of VSMC and the release of chemoattractant protein-1 (MCP-1). IL-6 increases the number of platelets in circulation, their modification and the level of fibrinogen and coagulant phase of the clotting mechanism that may lead to pathological thrombosis.^(26)^

Several recent clinical studies suggest a close association between serum CRP and ocular vascular disorders related to AMD.^(27–29)^ The findings of Nagaoka *et al.*^(30)^ suggested that detrimental effects of CRP could also affect the ocular circulation and might partially contribute to development of the retinal vascular disease. De Jong *et al.*^(31)^ showed in the Rotterdam Study, that there was a small, but significant association between CRP levels and AMD incidence. Kikuchi *et al.*^(32)^ demonstrated the trends of the increased risk of disease with the increase of CRP, what was statistically significant for both polypoidal choroidal vasculopathy (PCV) and neovascular AMD. The Rotterdam Study found that elevated baseline levels of high sensitive CRP were associated with the development of early and advanced AMD.^(33)^

However, Klein and colleagues demonstrated no significant association between CRP plasma concentration and AMD or AMD progression in both case-control and prospective studies.^(34)^ In a case-control study using patients recruited from Muenster (Germany), researcher found significantly elevated CRP levels as the degree of AMD severity increased compared with controls,^(35)^ but when cardiovascular risk factors were taken into consideration no statistically significant increases were found with ORs of AMD patients compared with controls.^(35)^

IL-6 is a marker for systemic inflammation. Seddon and colleagues performed a prospective cohort study with the aim to demonstrate if IL-6 could predict progression of AMD.^(36)^ The group found a correlation between the level of IL-6 and chances of AMD progression, and they prooved that elevated IL-6 may serve as marker for progression of AMD. However, Klein and colleagues found no significant association between plasma IL-6 levels and AMD, or AMD progression.^(34)^

Similar to our results, Cohen *et al.*^(37)^ proved that significant association existed between antioxidant enzymes and AMD. They found that subjects with lower GR (OR: 1.63, 95% IP: 1.0–8.0, *p* = 0.05) and GPx-a (OR: 1.36, 95% IP: 1.0–2.0), had higher chances to develop AMD compared to subjects with normal enzymes values. Evans^(38)^ went one step further, showing in their study (meta-analysis), that the imbalance between oxidants, and antioxidants which can physiologically occur with aging, can be repair with antioxidant supplementation, which can reduce the risk of developing AMD, and may even reduce the progression of late forms AMD and vision loss (OR: 0.77, 95% CI: 0.62–0.96). Yamato *et al.*^(39)^ showed that Tempol (4-hydroxy-2,2,6,6-tetramethyl piperidine-*N*-oxyl) drinking could reduce lipid peroxidation and CRP levels and increase the concentration of ascorbic acid in aged-mice compared to the young mice. This study documented that usage of a relatively low concentration (6 mM) of Tempol could have a life-extending effect, because of its impact on chronic inflammation and beneficial effects on the immune and antioxidant system.

A case-control study of Lip and colleagues recently found elevated levels of plasma fibrinogen in AMD cases compared to the controls.^(40)^ A case-control analysis from the large Blue-Mountaines Eye Study in Australia detected significantly elevated plasma fibrinogen levels in late AMD patients compared to the controls (*p*<0.05).^(41)^ The relative risk of late AMD was 6.7 for fibrinogen levels higher than 4.5 g/L (highest quartile) compared to the lowest quartile.^(41)^ In another study using patients recruited from the Muenster Aging and Retina Study population in Muenster, the researches found elevated plasma fibrinogen levels as the degree of AMD severity increased.^(34)^ Schaumberg *et al.*^(42)^ in his 10 years follow-up study on 275,000 subjects found a higher probability for AMD in subjects with higher hCRP (OR: 3.09, 95% IP: 1.39–6.88, *p* = 0.02) and fibrinogen values (OR: 2.01, 95% IP: 1.07–3.75) compared to subjects with normal values of these parameters.

Age-related impaired function of the antioxidant system has been reported by many studies. Like other age-related degenerative diseases, the incidence of AMD rises exponentially with aging. During aging and pathological conditions, the balance between the ROS generation and ROS clearance can be disturbed due to reduced antioxidant defense system resulting in oxidative damage to macromolecules. Therefore, the therapy with antioxidants and anti-inflammatory substances may be beneficial for improvement of an endothelial function and prevention of AMD.

## Figures and Tables

**Table 1:** Demographic and biochemical parameter values in the subgroups of AMD patients and control group subjects

Parameters	AMD Early (*n* = 31)	AMD Exudative (*n* = 75)	CG (*n* = 84)	*p*
Age (years)	74.15 ± 6.3*	70.49 ± 7.1*	70.47 ± 4.18	**0.019**
Gender (M/F)	35.5/64.5	22.7/77.3	48/52	**0.027**
Smokers (%)	37.3	46.9	41.4	0.675
SOD (IU/gHb)^‡^	1,169 ± 173*	1,081.6 ± 170.3^♦^	1,079.3 ± 128.3	0.06
GPx (IU/gHb)^‡^	40.1 ± 10.8*	34.5 ± 10.0	39.9 ± 9.66	**0.025**
GR (U/L)^†^	51 (46–59)	63 (46–72)^♦^	59 (54–64.5)	0.07
Albumin (g/L)^‡^	43.6 ± 2.6*	42.8 ± 2.19*^♦^	44.8 ± 2.8	**0.001**
Uric acid (µmol/L)^†^	270 (211.8–288.5)*	272 (219.5–316.5)*	295.5 (248–350)	**0.006**
Bilirubine (µmol/L)^†^	9.7 (7.8–13.2)	11.2 (8.2–13.7)	11.5 (9.0–14.3)	0.430
TAS (mmol/L)^‡^	1.18 ± 0.14*	1.02 ± 0.22*	1.31 ± 0.17	**<0.000**
CRP (mg/L)^†^	2.4 (1.63–7.75) ^♦^	3.65 (2.10–6.10)*	3.0 (1.65–4.10)	**0.05**
IL-6 (pg/ml)^†^	2.82 (1.68–3.88)	3.54 (1.57–15.89)*	2.30 (1.52–3.48)	0.07
Fibrinogen (g/L)^†^	3.91 (3.3–4.0)	3.98 (3.3–4.6)*	3.5 (3.0–4.0)	**0.007**

AMD, The patients with Age-related Macular Degeneration; CG, the control group. ^‡^Values are presented as Arhitmethic mean ± SD. ^†^Values are presented as Median and interquartile range. *p<0.05, difference between the tested group and the control group. ^⧫^*p*<0.05, difference between the early and late-exudative AMD group. *P*, significance of difference.

**Table 2:** Logistic regression analysis: the association of tested parameters with the exudative form of AMD (χ^2^ = 27.3, *p* = 0.0003)

Variable	Odds ratio	95% CI	*p*
CRP	1.16	1.03–1.32	**0.018**
Fibrinogen	2.31	1.14–4.85	**0.021**
IL-6	1.09	0.93–1.27	0.283
GPx	1.01	0.98–1.04	0.575
GR	0.98	0.94–1.03	0.408
SOD	1.001	0.998–1.002	0.928
TAS	7.45	3.97–14.35	**<0.001**
Albumine	1.25	1.11–1.41	**0.0001**
Uric acid	1.006	1.00–1.02	**0.003**
Total bilirubine	0.217	0.92–1.03	0.376

95% CI, 95% confidence interval; *p*, significance of difference.
